# Retraction Note: Hsa_circ_0005379 regulates malignant behavior of oral squamous cell carcinoma through the EGFR pathway

**DOI:** 10.1186/s12885-023-11178-6

**Published:** 2023-07-17

**Authors:** Wen Su, Yufan Wang, Feng Wang, Shuai Sun, Minghua Li, Yuehong Shen, Hongyu Yang

**Affiliations:** 1 Department of Oral and Maxillofacial Surgery, Peking University Shenzhen Hospital, No. 1120 Lianhua Road, Shenzhen, 518001 Guangdong China; 2 Peking University Shenzhen Hospital Clinical College, Anhui Medical University, Hefei, Anhui China; 3 Central laboratory, Peking University Shenzhen Hospital, Shenzhen, Guangdong China

**Retraction Note: BMC Cancer 19, 400 (2019)**

**https://doi.org/10.1186/s12885-019-5593-5**

The Editor has retracted this article at the Corresponding Author's request. After publication, concerns were raised regarding highly similar images used in this and other articles, specifically:

- In Fig. 2b, the circRNA overexpression group images appear highly similar to the Control group in Fig. 2d of ([1], now retracted);
- Figs. 4c, 4d and 8f appear to contain overlapping panels with Figs. 4d, 4e, 7e and 7f of [2];
- In Fig. 5a, Mock SCC25 and CAL27 appear to contain an overlapping area.
- In Fig. 5b, Mock SCC25 and CAL27 appear to original from the same sample, with rotation and minor modification.

The authors have stated that these images were misused. The Editor therefore no longer has confidence in the presented data.

Wen Su, Feng Wang, Yuehong Shen and Hongyu Yang agree to this retraction. Yufan Wang, Shuai Sun and Minghua Li have not responded to any correspondence from the editor or publisher about this retraction.
